# Vitamin D deficiency and its impact on asthma severity in asthmatic children

**DOI:** 10.1186/s13052-016-0300-5

**Published:** 2016-12-17

**Authors:** Nasrin Esfandiar, Fariba Alaei, Shahrzad Fallah, Delara Babaie, Niloofar Sedghi

**Affiliations:** 1 Pediatric Nephrology Research Center, Faculty of Medicine, Mofid Children’s Hospital, Shahid Beheshti University of Medical Sciences, Tehran, Iran; 2 Department of Pediatrics Cardiology, Faculty of Medicine, Mofid Children’s Hospital, Shahid Beheshti University of Medical Sciences, Tehran, Iran; 3 Department of Emergency Medicine, Faculty of Medicine, Mofid Children’s Hospital, Shahid Beheshti University of Medical Sciences, Tehran, Iran; 4 Allergy and Clinical Immunology Department, Mofid Children’s Hospital, Shahid Beheshti University of Medical Sciences, Tehran, Iran

**Keywords:** Asthma, Vitamin D, Children, Risk

## Abstract

**Background:**

Despite obtaining evidences on association between vitamin D and development of lung in fetus, little is known about vitamin D level and its impact on severity of asthma in children. The present study aimed to assess the relationship between the asthma severity and vitamin D deficiency in asthmatic children.

**Methods:**

This case-control study was conducted on 106 individuals including asthmatic (*n* = 53) and healthy children (*n* = 53) who referred to Mofid hospital in Tehran in 2013. The level of serum vitamin D in both groups was measured by radioimmunoassay method at the reference lab and was categorized as sufficient (> 30 ng/ml), insufficient (20 to 30 ng/ml), or deficient (< 20 ng/ml). The control status of asthma in patients group was classified as controlled, partially controlled, and uncontrolled.

**Results:**

In the groups with and without asthma, the prevalence of vitamin D deficiency was 73.6 and 49.1%, and the prevalence of vitamin D insufficiency was 18.9 and 18.9%, while normal vitamin D level was revealed in 7.5 and 32.1%, respectively with a significant difference (*p* = 0.005). Using the multivariate logistic regression analysis, the presence of asthma was associated with reduced level of vitamin D (OR = 1.068, 95% CI: 1.027–1.110, *P* = 0.001). In this context, the risk for asthma in the children with vitamin D deficiency was 6.3 times of those with normal vitamin D level. Although the presence of asthma was strongly associated with reduced level of vitamin D in serum, neither severity of asthma nor control status of asthma were associated with vitamin D deficiency.

**Conclusion:**

The presence of vitamin D deficiency effectively predict increased risk for childhood asthma; however the severity or control status of this event may not be predicted by confirming vitamin D deficiency.

## Background

The upward trend of morbidity and high socioeconomic burden of asthma in children has prompted scientists to seek both genetic and environmental factors contribute to this phenomenon. Recently, the relationship between vitamin D deficiency and pediatrics asthma has been supported by some clinical studies [1]. Recent studies introduced a genetic factor, vitamin D receptor gene polymorphisms, that is responsible for vitamin D deficiency in children. In this regard, the predictive role of some polymorphisms such as FokI, ApaI, and TaqI has been suggested, but not completely understood [2]. Also, in a developing long time series transcriptome data (DLCGS) in order to infer the role of in utero changes of vitamin D responsive genes in both the developing lung and asthma, some evidences have been obtained the set of vitamin D related genes to be associated with lung development [3], but these gene associations have not been clearly determined in other population-based studies. On the other hand level of pH in exhaled breath condensate decrease in asthma exacerbations [4], and vit-D deficiency aggravates oxidative stress and DNA damage [5]. Clinically, the association between vitamin D and fetal lung development has been also revealed in both animal and fetal models. In animal studies, it has been found that rachitic rat pups born to vitamin D deficient mothers had reduced lung compliance and also delayed alveolar development [4, 6, 7]. It has been also shown vitamin D3 as a main growth factor necessary for proliferation of alveolar type-II cells and the vitamin D receptors has been characterized in alveolar epithelial cells [8]. Additionally, it has been revealed that exposure to vitamin D in fetus leads to increase in surfactant synthesis and secretion [9–11]. There is an important point that vitamin D deficiency is a common finding among children and young adolescent whole of the world especially in our country. In a study among Iranian young students, more than half of them suffered vitamin D deficiency [12]; however, we could not find a comprehensive data on the prevalence of vitamin D deficiency among Iranian infants. Despite obtaining evidences on association between vitamin D and development of lung in fetus, little is known about vitamin D levels and their impact on severity of asthma in children. Hence, we hypothesized that the children with severe asthma had lower level of vitamin D. The present study aimed to assess the relationship between the severity of asthma and severity of vitamin D deficiency in asthmatic children.

## Methods

This case-control study was conducted on 53 asthmatic patients and 53 healthy controls in Mofid hospital, Tehran during 2013. The selection of samples was based on simple census sampling method. The patients aged above 2 years who were diagnosed with asthma and hyperreactive airway disease, were included in the study. Asthma was diagnosed according to the EPR3 and GINA criteria [1, 13]. All included patients gave informed written consent and the study was approved by the ethics committee at Shahid Beheshti University of Medical Sciences (registration number: IR.SBMU. MSP.REC.1392.405). Human rights were respected in accordance with the Helsinki Declaration. The exclusion criteria were having disease or conditions which might affect serum level of vitamin D and or its metabolism, including chronic renal failure, metabolic disorder, malabsorption, cholestasis, refractory Rickets.

The control subjects were randomly selected from children without asthmatic children or other underlying disorders the baseline characteristics were collected from clinical checklist completed by interviewing parents and patients including gender, age, duration of asthma, and hospitalization rate. The level of serum vitamin D in both groups was measured by radioimmunoassay (RIA) method at the reference lab and was categorized as sufficient (> 30 ng/ml), insufficient (20 to 30 ng/ml), or deficient (< 20 ng/ml). The severity of asthma was also stratified according to the asthma guideline into four grades of intermittent or mild, moderate, severe persistent [14]. Also, the control status of asthma in patients group was classified as controlled, partially controlled, and uncontrolled based on GINA (Global Initiative for Asthma) criteria including daytime symptoms, limitation of activities, nocturnal symptoms, need for reliever or rescue inhaler, and lung function [13]. Moreover, the risk for appearance of symptoms was assessed based on the frequency of worsening symptoms need to use systemic corticosteroids according to the guidelines [1, 13].

For statistical analysis, Results were presented as mean ± standard deviation (SD) for quantitative variables and were summarized by absolute frequencies and percentages for categorical variables. Categorical variables were compared using chi-square test or Fisher's exact test when more than 20% of cells with expected count of less than 5 were observed. Quantitative variables were also compared with *t* test, ANOVA (Analysis of Variance) tests or Mann- Whitney *U* test. The association between quantitative variables was tested using the Pearson’s or Spearman’s correlation test. We used the multivariable regression modeling to assess the relation between vitamin D deficiency and severity of asthma with the presence of confounders. For the statistical analysis, the statistical software SPSS version 16.0 for windows (SPSS Inc., Chicago, IL) was used. *P* values of 0.05 or less were considered statistically significant.

## Results

**Fig. 1:**
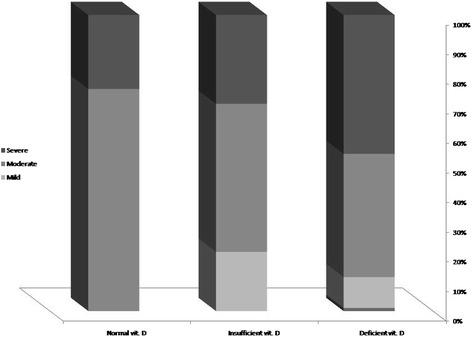
The association between vitamin D deficiency and severity of asthma

**Fig. 2:**
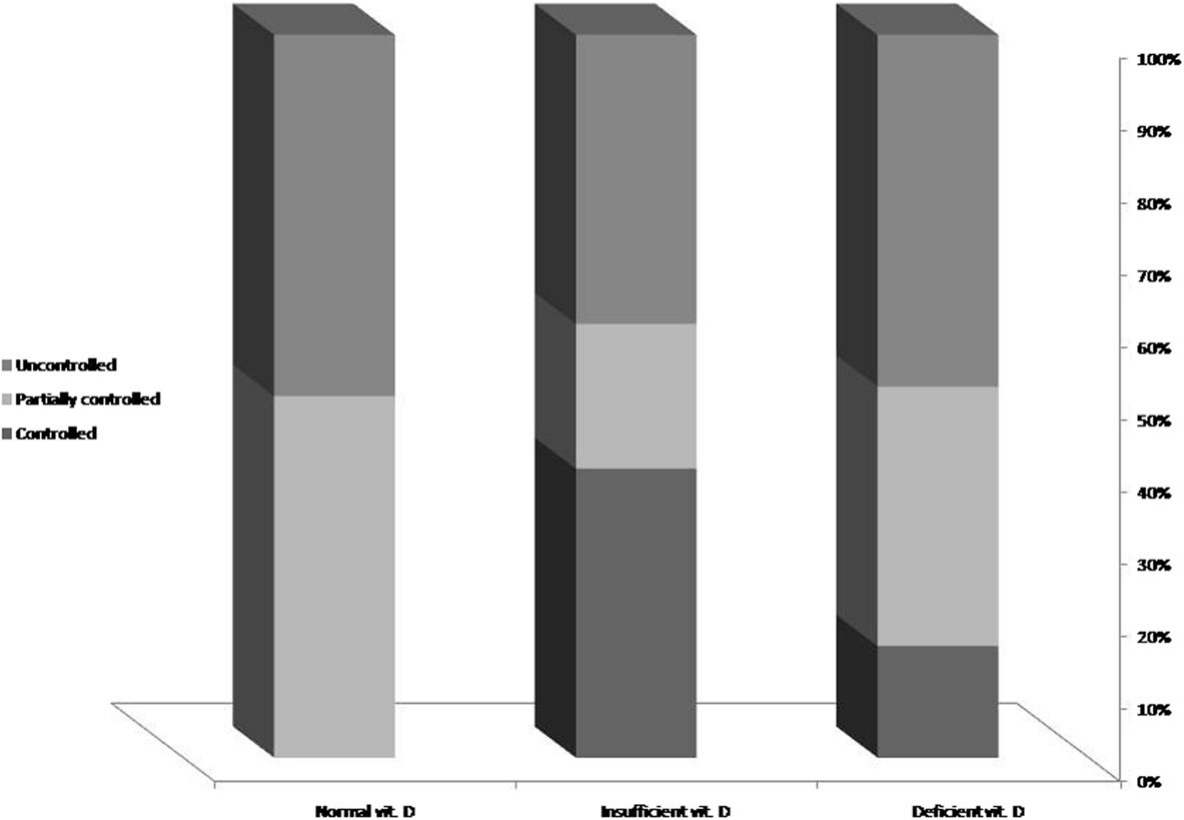
The association between vitamin D deficiency and control status of asthma

**Fig. 3:**
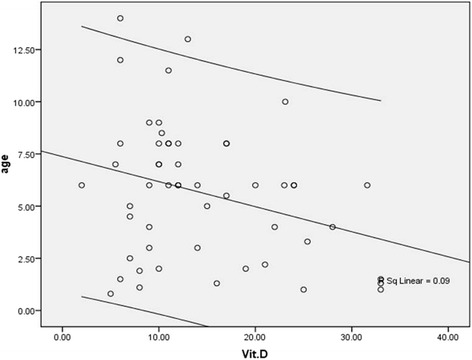
Correlation between level of vitamin D and patients’ age

The two groups were similar in male gender distribution (56.6 versus 59.0%, *p* = 0.559) and mean age (5.63 ± 3.24 years versus 5.56 ± 3.90 years, *p* = 0.920, which 43.4% in the case group were women and 49.1% in the control group as well) (Table 1). In asthmatic group, the mean duration of disease was 13.1 ± 24.6 months. Regarding rate of admission, 13.2% of patients were hospitalized once, 9.4% of patients were hospitalized twice, and 1.9% of them were also hospitalized three times. In the patients group and regarding the severity of asthma, 11.3% were classified as mild asthma, 45.3% as moderate asthma, and 43.4% as severe asthma. Also regarding control status of asthma, the disease was controlled in 18.9%, partially controlled in 34.0%, and uncontrolled in 47.2%. According to the the level of disease attack risk, 5 of 31 children aged less than 5 years (16.1%) classified as high-risk, while 7 of 22 children older than 5 years (31.8%) classified as high risk. Assessing the serum level of vitamin D showed that the asthmatic children had significantly lower level of vitamin D compared to normal cases (14.53 ± 8.10 ng.ml versus 22.45 ± 13.46 ng/ml, *p* < 0.001). Also, in the groups with and without asthma, the prevalence of vitamin D deficiency was 73.6 and 49.1%, and the prevalence of vitamin D insufficiency was 18.9 and 18.9%, while normal vitamin D level was revealed in 7.5 and 32.1%, respectively with a significant difference (*p* = 0.005). As summarized in Figs. 1 and 2, despite the presence of asthma was strongly associated with reduced level of vitamin D in serum, but neither severity of asthma nor lack of controlling disease was associated with vitamin D deficiency. In this regard, mean serum level of vitamin D in the patients with mild asthma was 12.85 ± 7.06 ng/ml, in the group with moderate asthma was 16.98 ± 8.63 ng/ml, in the patients with severe asthma was 12.42 ± 7.52 ng/ml, and in those with persistent asthma was 12.01 ± 5.00 ng/ml (*p* = 0.260). Furthermore, the mean serum level of vitamin D in children with controlled asthma was 15.11 ± 8.22 ng/ml, in the group with partially controlled asthma was 15.56 ± 7.88 ng/ml, and in those with uncontrolled asthma was 13.55 ± 8.42 ng/ml with no difference (*p* = 0.711). Among children younger than 5 years, no difference was found in low-risk and high-risk subgroups for disease attacks in mean level of vitamin D (14.40 ± 10.54 ng/ml versus 14.53 ± 7.72 ng/ml, *p* = 0.976), while in older group, the level of serum vitamin D was significantly lower in the high-risk group than in low-risk group (9.21 ± 3.08 ng/ml versus 17.05 ± 8.99 ng/ml, *p* = 0.038). There was no difference in mean level of vitamin D in male and female asthmatic patients (15.54 ± 8.79 ng/ml versus 13.20 ± 7.07 ng/ml, *p* = 0.303), whereas an adverse correlation was found between patients’ age and level of vitamin D (*r* = −0.300, *p* = 0.029) (Fig. 3). No association was revealed between duration of disease and level of vitamin D (*r* = −0.155, *p* = 0.267). Also, level of vitamin D was not associated with the frequency of hospitalization (*r* = −0.125, *p* = 0.274). As shown in Table 2 and using the multivariate logistic regression analysis, the presence of asthma was associated with reduced level of vitamin D (OR = 1.068, 95% CI: 1.027–1.110, *P* = 0.001). In this context, the risk for asthma in the children with vitamin D deficiency was 6.3 times of those with normal vitamin D level.

**Table 1:** Patients characteristics in two groups

	Case	Control	*P*-value
Age	5.6 ± 3.2	5.5 ± 3.9	0.902
Female (%)	23 (43.4%)	26 (49.1%)	0.349
Male	27 (56.6%)	30 (59.0%)	0.559
Vitamin D	14.5 ± 8.1	22.4 ± 13.4	0.0001

**Table 2:** Multivariate logistic regression analysis to determine association between asthma and serum level of vitamin D

	B	S.E.	Wald	df	Sig.	Exp (B)	95.0% C.I.for EXP (B)	
							Lower	Upper
Vit.D	.065	.020	10.805	1	.001	1.068	1.027	1.110
sex	.240	.418	.330	1	.566	1.272	.560	2.887
age	.000	.061	.000	1	.987	.999	.887	1.125
Constant	−1.527	.788	3.754	1	.053	.217		

## Discussion

The main point of the study was that the presence of vitamin D deficiency could effectively predict increased risk for childhood asthma; however, the severity or control status of this event could not be predicted by confirming vitamin D deficiency. Some case-controlled studies could show the association between vitamin D deficiency and asthma in children. In a study by Arikoglu et al. [15], the association between vitamin D deficiency and increased risk for asthmatic attack in children was indicated. In this regard, the mean serum level of vitamin D in the asthma attack group was significantly lower than that of the controlled asthma group. They also showed that the reduced vitamin D could increase the risk for asthma attack by 16 times, however we could not demonstrate relationship between vitamin D deficiency and asthma attack in our children. In another study by Hatami et al. [16] and similar to our finding, there was a significant decrease in the concentration of serum 25- hydroxy (OH) vitamin D in the asthmatic patients as compared. Also, 56% of children were vitamin D deficient that was lower than that revealed in our study as 73.6%. In a review on observational studies including 3 prospective, 16 case-control and 14 cross-sectional studies, it was suggested a pooled positive association of vitamin D levels with better asthma control, reduced use of asthma medication, fewer asthma exacerbations and lower utilization of health care facilities for urgent treatment [17]. Even, some clinical trials structured as a systematic review demonstrated that the use of supplement vitamin D could be effective in reducing the risk for asthma progression. In a meta-analysis by Riverin et al. [18] in 2015 and by reviewing eight randomized controlled trial including 573 children aged 3 to 18 years; it was revealed that the use of vitamin D supplement led to a reduced risk of asthma exacerbations with the relative risk of 0.41. In this regard, the serum 25-hydroxy vitamin D level was higher in the vitamin D group at the end of the intervention. In total, comparing our study with the previous reports shows two important points. First, according the reports on prevalence of vitamin D deficiency among our children, most Iranian children suffer vitamin D deficiency that even in our control group; about half of the children in different geographical areashave vitamin D deficiency. In a study by Saki et al. [19] in southern Iran, shocking statistics was reported from vitamin D among children so 81.3% of them were vitamin D deficient. In another survey by Kelishadi et al. [20] in Isfahan province, vitamin D deficiency and insufficiency were detected in 37.9 and 46.3% of children, respectively. Second, the reports found higher risk of vitamin D deficiency for asthmatic children when compared to general population emphasizing administration of supplement vitamin D in these children subgroup.

Some mechanisms have been introduced to explain association between vitamin D deficiency and progression of asthma especially in infants. One mechanism explained focus regulatory role of vitamin D in immune system functions. The oxidative stress plays a major role in asthma exacerbations (4). Vitamin D deficiency enhances oxidative stress (5). Vitamin D deficiency was shown to be effective on Th1 and Th2 cytokines secretion, which can contribute to the development of atopy. In fact, neonates with vitamin D deficiency develop a Th2-skewed pulmonary immune phenotype and reduced IL-10-secreting T regulatory cells affecting neonatal pulmonary function [20–22]. Vitamin D Receptor (VDR) activation inhibits IgE expression in B cells and enhances IL-10 expression, which studies show can protect against atopic conditions [23–25]. Besides, it has been shown that 1,25-dihydroxycholecalciferol, the active form of vitamin D, is a paracrine factor that modulates fetal lung maturation and airway smooth muscle cell proliferation and differentiation [26, 27]. In one survey D supplementation associated with asthma controllers could significantly improve FEV_1_ in mild to moderate persistent asthma after 24 weeks (2).

Therefore, it seems that using vitamin D3 supplementation can be effective on blocking the increase in tracheal contractility that developed in the vitamin D deficient children.

## Conclusion

In summary, vitamin D deficiency and insufficiency is a common finding among asthmatic children that may be associated by the physiological mechanism of this phenomenon. In fact, it seems that the use of supplement vitamin D can prevent progression of childhood asthma. Providing vitamin D in children especially among Iranian people with high prevalence of vitamin D deficiency can be very helpful to prevent upward trending severe asthma among children with vitamin D deficiency. Further longitudinal studies on the effects of vitamin D supplementation in childhood asthma control would help understanding real life outcomes.

## Abbreviations

ANOVA: Analysis of variance
GINA: Global initiative for asthma
OH: Hydroxy
RIA: Radioimmunoassay
VDR: Vitamin D receptor
EPR3: Expert panel report 3
